# A Huge Conjunctival Atypical Fibroxanthoma

**DOI:** 10.7759/cureus.71130

**Published:** 2024-10-09

**Authors:** Estefania Ramirez Marquez, José J López-Fontanet, Gerardo Torres, Jean Lafontaine, Maria Correa, Armando L Oliver, Joseph Campbell

**Affiliations:** 1 Ophthalmology, University of Puerto Rico School of Medicine, Medical Sciences Campus, San Juan, PRI; 2 Radiology, University of Puerto Rico School of Medicine, Medical Sciences Campus, San Juan, PRI; 3 Pathology and Laboratory Medicine, University of Puerto Rico, Medical Sciences Campus, San Juan, PRI; 4 Ophthalmology, University of Puerto Rico, Medical Sciences Campus, San Juan, PRI

**Keywords:** atypical fibroxanthoma, conjunctiva, cornea, exenteration, tumor

## Abstract

We report the case of a Hispanic male whose conjunctival atypical fibroxanthoma (AFX) grew very large, with intraocular as well as extraocular muscle extensions, and was treated with exenteration. A 50-year-old male presented with a one-month history of foreign-body sensation in his left eye. The initial examination revealed an erythematous, vascularized, pedunculated lesion arising from the left eye conjunctiva. A tissue biopsy from the lesion confirmed the diagnosis of an AFX with a small component of squamous cell carcinoma. The patient was intermittently lost to follow-up until, one-and-a-half years after his initial visit, he underwent an exenteration of the left eye as the tumor had intraocular and extraocular muscle extensions. Subsequently, he was scheduled for ongoing monitoring.

## Introduction

An atypical fibroxanthoma (AFX) is a rapidly growing lesion with a fleshy appearance and an apparent predilection for sun-exposed tissues [1-5]. The diagnosis is based on histologic findings, including pleomorphism, atypical mitotic figures, and spindle architecture [1,4]. This lesion is most commonly found on the skin of the heads and necks of elderly Caucasian patients [2,4,5]. The prognosis for an AFX is mostly positive as the lesions are readily treated with surgical excision and rarely recur [4,5].

There are limited reports of AFXs involving ocular structures such as the conjunctiva, cornea, and ocular muscles [1-5]. Additionally, the coexistence of AFX and squamous cell carcinoma has been documented in the literature as collision tumors [6]. Here, we present the case of a Hispanic male whose conjunctival AFX grew very large, with intraocular as well as extraocular muscle extensions, and was treated with exenteration.

## Case presentation

A 50-year-old Hispanic male presented with a one-month history of foreign-body sensation in his left eye (OS). His ocular and systemic medical history was unremarkable. His review of systems, as well as his past social and family histories, were otherwise unremarkable. The patient delayed seeking treatment because he had associated his complaint with an incident in which cement particles had entered his eyes. After that event, he received treatment with an unspecified medication that provided relief. However, the day before arriving at the emergency room, he noticed that the lesion in his eye was protruding and had begun bleeding.

**Figure 1:**
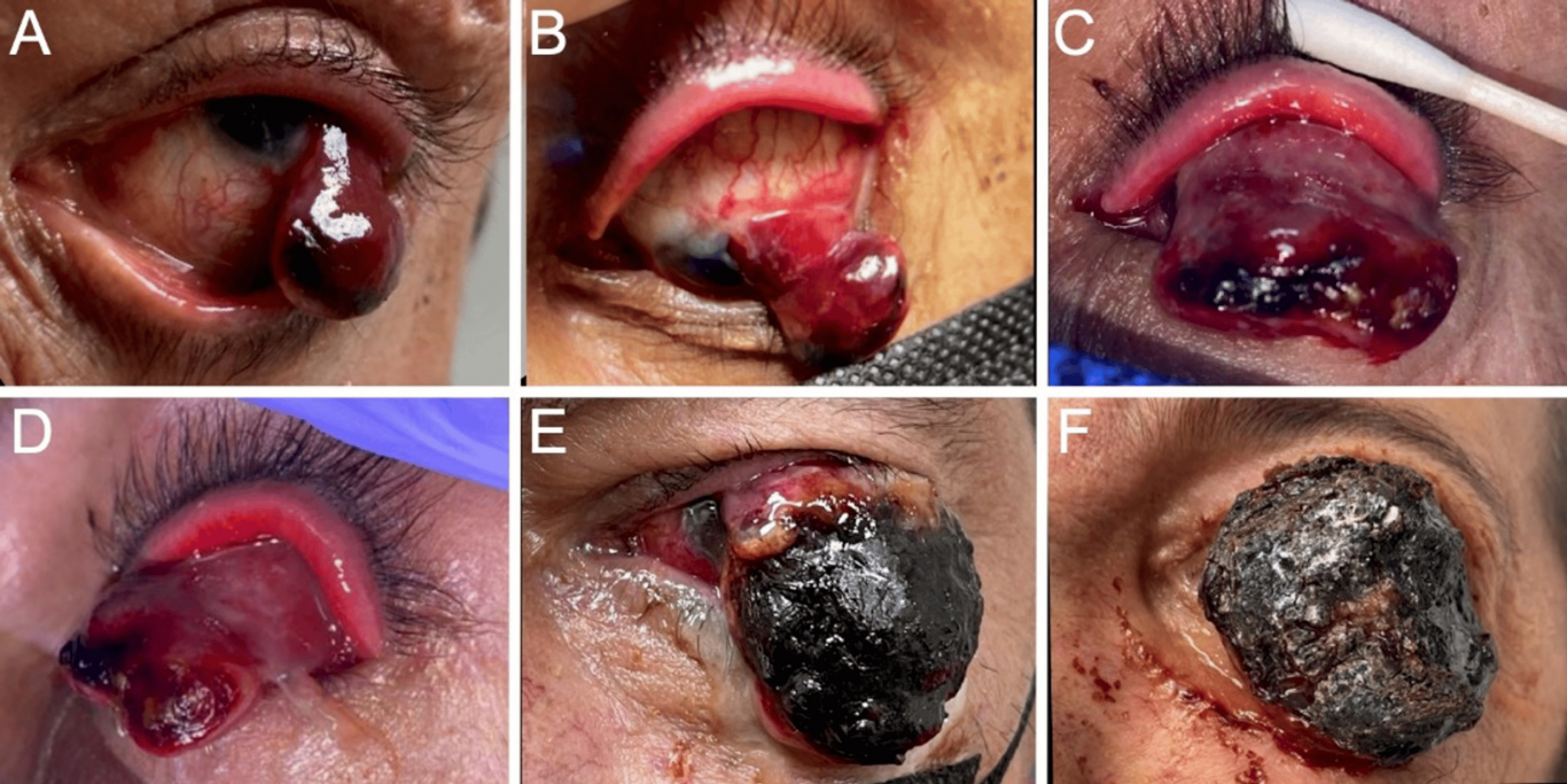
Photographs of the progression of the patient’s left-eye atypical fibroxanthoma. External photographs of the patient upon initial evaluation reveal (A) a hemorrhagic and pedunculated lesion prolapsing from the conjunctiva and (B) a vascular pedunculated mass extending from the bulbar conjunctiva with prominent feeder vessels and distal ulceration. Photographs three months later show a (C) front and (D) lateral view of a hemorrhagic mass with a purple hue obscuring the view of the ocular surface and distal ulcerative changes. After 10 months, photographs reveal (E) a large pedunculated mass with distal ulcerated and necrotic changes originating from the bulbar conjunctiva and cornea. Photograph 16 months after the initial encounter during (F) preoperative evaluation reveals a large pedunculated mass with superior hemorrhagic and purulent tissue.

Upon a comprehensive ophthalmic evaluation, his best-corrected visual acuity (BCVA) was 20/20 in the right eye (OD) and 20/50 OS. The intraocular pressure was 9 mmHg OD and 6 mmHg OS. The pupils were round and reactive to light, and there was no afferent pupillary defect. Color vision, as assessed by the Ishihara color plate test, revealed no defect in either eye. Extraocular movements were within normal limits in both eyes (OU). Gonioscopy was unremarkable OU. A slit-lamp examination (SLE) revealed an erythematous, vascularized, and pedunculated lesion arising from the bulbar conjunctiva (OS) (Figure 1, Panels A and B). The right eye was unremarkable. The patient’s fundus was unremarkable OU.

**Figure 2:**
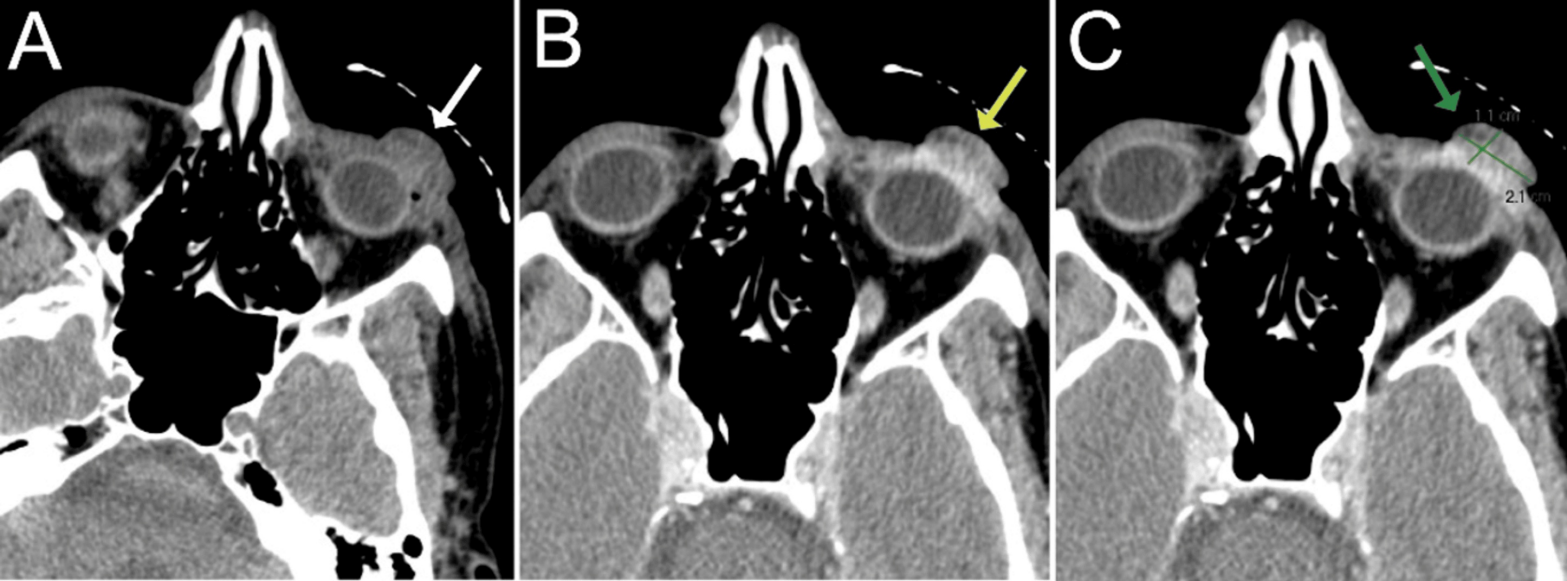
Axial orbital computed tomography scans. (A) Pre-contrast and (B) post-contrast scans reveal an exophytic-enhancing conjunctival lesion around the inferior aspect of the anterior left globe measuring approximately 1.1 cm (anterior-posterior) × 2.1 cm (transverse) × 1.7 cm (craniocaudal). There was no involvement of the eyelid, lacrimal gland, extraocular muscles, or left lens.

**Figure 3:**
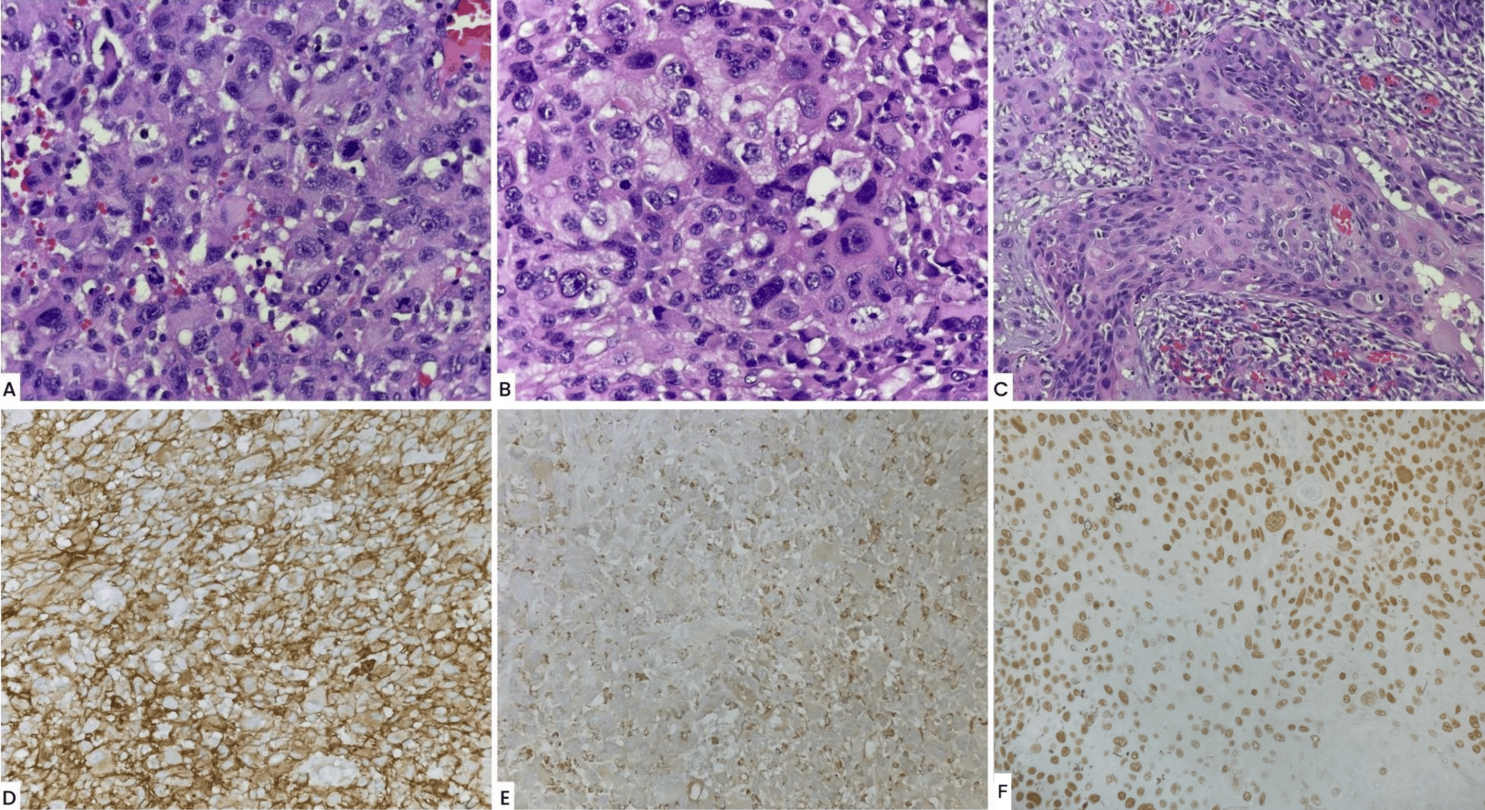
Histopathological images. (A) Pleomorphic cells with hyperchromatic nuclei and abundant eosinophilic cytoplasm and numerous mitotic figures (hematoxylin and eosin (H&E), 40×). (B) Higher magnification showing pleomorphism, hyperchromasia, prominent nucleoli, and bizarre multinucleated giant cells (H&E, 60×). (C) Squamous cell component. (D) CD10, strong and diffuse immunoreaction (20×). (E) CD68 positivity (20×). (F) p40 positivity (40×).

The patient was prescribed neomycin/polymyxin B/dexamethasone ophthalmic ointment (3.5 mg/10,000 units/1 mg/g, four times daily), OS, and scheduled for a follow-up the next day. However, he did not return until three months after his initial presentation, when he was rereferred to the clinics with a suspicion of a ruptured globe. His BCVA had decreased to light perception OS. An SLE was remarkable (OS) for upper and lower lid tenderness as well as a vascular, pedunculated, prolapsing lesion with necrotic tissue, anteriorly, and superior prominent feeder vessels with suspected deep stromal infiltration (Figure 1, Panels C and D). Brightness scan ultrasonography revealed no evidence of globe rupture or prolapse. An orbital computed tomography scan revealed diffuse left orbital preseptal swelling with an intact eye globe (Figure 2). A biopsy was consistent with the diagnosis of AFX, with a focal component of SCC. AFX tumor disclosed immunoreaction to CD10 and CD68, while the SCC component was confirmed by positivity to cytokeratins CK7 and CK5/6, in addition to positivity to EMA, p40, and p63. The exanteration specimen revealed similar histopathologic findings and immunohistochemistry profiles (Figure 3). Additional immunostains performed in biopsy ruled out melanoma and other sarcomas with similar histopathologic features considered in the differential diagnosis, such as pleomorphic spindle cell SCC, leiomyosarcoma, malignant fibrous histiocytoma, and angiosarcoma, among others. The presence of AFX and SCC has been described as collision tumors, that is, two different tumors with distinct borders. This phenomenon is well demonstrated in the resection specimen.

The patient was scheduled for a diagnostic and debulking surgery; nevertheless, he was lost to follow-up for an additional 10 months. Upon re-evaluation, his BCVA had decreased to no light perception, and his ocular movements were restricted in all directions OS. An external examination revealed that the mass was ulcerated and had mucus secretions (Figure 1, Panel E). An SLE examination was not possible as the mass occluded the vision of all the other structures of the globe OS.

Sixteen months after the patient’s initial visit, he underwent an exenteration OS as the tumor had intraocular and extraocular muscle extensions (Figure 1, Panel F). The left orbit exenteration revealed an exophytic ulcerated tumoral mass involving the entire conjunctiva with no discernible eye structures. The tumor measured 3.5 × 3.5 × 1.5 cm and presented a 0.3 cm rim of the eyelid, which appeared grossly negative for the tumor. The tumor was tan brown and presented an irregular contour with focal areas of hemorrhage. On section, the eyeglobe was not involved by the tumor. Microscopically, the tumor had a similar histology as the initial biopsy, with sheets of highly atypical and pleomorphic cells, some with an epithelioid appearance and prominent nucleoli (Figure 3, Panels A and B). The cytoplasm of tumor cells was abundant, eosinophilic, and sometimes foamy or vacuolated. Scattered large, bizarre-appearing multinucleated cells were also seen as well as numerous mitotic figures. The immunophenotype was similar as well. The margins of resection, including the optic nerve margin, were negative for the tumor. A separate tumor fragment was also received that measured 4.4 × 3.5 × 1.3 cm and presented a similar macroscopic and microscopic appearance.

He was scheduled for a postoperative follow-up, and two weeks after his surgery was found to be healing well, with healthy granulation tissue and no evidence of infection or recurrence.

## Discussion

To our knowledge, this case is one of the largest AFXs documented to have involved ocular tissue. The conjunctiva and cornea constitute rather rare locations of an AFX [1,4,5]. A patient with an AFX typically presents a 1-2 cm, vascular, dome-shaped lesion on the skin of the head or neck; such lesions tend to range from flesh-colored to red and have areas of bleeding or ulceration [2-4]. This condition is a variant of undifferentiated pleomorphic sarcoma; however, it tends to behave less aggressively [2,4]. Histopathologically, an AFX is distinguished by the presence of atypical pleomorphic spindle cells with hyperchromatic nuclei and abundant cytoplasm [1,2]. Cases with tumor necrosis, invasion into subcutis, and/or lymphovascular invasion have metastatic potential and should, therefore, be regarded as pleomorphic dermal sarcoma [7].

Possible risk factors for the development of an AFX include having suffered previous ocular trauma, having a history of extensive exposure to ultraviolet radiation, and having or having had any one or more of several diseases or disorders that predispose patients to tumorigenesis, such as xeroderma pigmentosum [1,2]. Our patient associated his condition with an event in which cement particles got in his eyes. It is also worth noting that he had had extensive lifelong exposure to ultraviolet radiation from the sun as he resides in a tropical area. It is possible that these elements had served as risk factors for the development of or had simply exacerbated an existing AFX lesion.

When an AFX affects the ocular tissues, its optimal management remains uncertain due to its rarity and the limited availability of data [4]. According to the current literature, the prognosis of an eye-involved AFX is almost entirely positive, so long as the lesion is biopsied and excised without further damage to the globe [1,5]. Nevertheless, the intraocular and extraocular muscle extension observed in this patient’s lesion warranted an exenteration. This case emphasizes the need for the timely and aggressive management of an AFX involving ocular tissues, as the disease may lead to the devastating loss of both vision and the eye globe. Further studies should be conducted to promote the development of improved algorithms for treating the ocular involvement of an AFX and thus preventing significant visual impairment.

## Conclusions

AFX is rarely found in eye tissues such as the cornea or conjunctiva. Nevertheless, this possibility should be considered in patients with rapidly growing, vascular, dome-shaped lesions. An accurate diagnosis requires histologic analysis. Timely attention and intervention are vital to avoid the sight and globe-threatening implications associated with this condition.
